# Long-term Outcomes in Anomalous Left Coronary Artery From the Pulmonary Artery Syndrome: A Multicenter Half-Century Experience

**DOI:** 10.1016/j.cjcpc.2025.07.005

**Published:** 2025-08-13

**Authors:** Sean D. Smith, Eiad Habib, Tala B. Shahin, Amani Elshaer, Claire Yee, Elaina A. Blickenstaff, Francois Marcotte, Alexander Egbe, Heidi M. Connolly, Joseph Dearani, Hartzell V. Schaff, David S. Majdalany

**Affiliations:** a Department of Cardiovascular Medicine, Mayo Clinic, Scottsdale, Arizona, USA; b Department of Internal Medicine, Mayo Clinic, Scottsdale, Arizona, USA; c Department of Cardiovascular Medicine, Mayo Clinic, Rochester, Minnesota, USA; d Department of Cardiovascular Surgery, Mayo Clinic, Rochester, Minnesota, USA

**Keywords:** congenital cardiac, anomalous, pulmonary artery

## Abstract

**Background:**

Anomalous left coronary artery from the pulmonary artery (ALCAPA) syndrome is a congenital anomaly that presents in infancy or rarely in adulthood and generally requires surgical correction. Data on long-term sequelae are minimal.

**Methods:**

All patients with ALCAPA seen within our hospital system from 1965 to 2022 were reviewed. Patients with other structural heart diseases were excluded.

**Results:**

Thirty-one patients were identified, with 9 patients (29.0%) being diagnosed as adults. The cohort was divided into pediatric (<18 years) and adult (≥18 years) subgroups. Heart failure was the most common presentation in pediatric patients (36.4%), whereas dyspnea and chest pain were more common in adults (44.4% and 33.3%, respectively). All patients underwent surgical repair. Overall survival was 93.4% at 1 year and 5 years (95% confidence interval [CI]: 85-100) and 83.6% at 10 years (95% CI: 69.8-100). The need for reintervention was 0% at 1 year, 15.8% at 5 years (95% CI: 0-31), and 29.8% at 10 years (95% CI: 3.4-49). Preoperative ejection fraction (left ventricular ejection fraction [LVEF]) was 51% for pediatric patients and 43% for adults; follow-up LVEF was 61% among pediatric patients and 55% among adults. Significant mitral valve regurgitation (MR) was noted on the preoperative echocardiogram in 30.8% of pediatric patients and 42.9% of adults. The prevalence of MR decreased to 11.8% in pediatric patients, but it was still 40% in adults at long-term follow-up.

**Conclusion:**

ALCAPA has variable presentations in childhood and adulthood. Overall survival after surgery is excellent. LVEF improved throughout the cohort and MR improved in pediatric patients. Reintervention rates increased over time.

## Résumé

**Contexte:**

L’anomalie de naissance de la coronaire gauche à partir de l’artère pulmonaire (ANCG) est une anomalie congénitale dont les manifestations surviennent généralement durant l’enfance, plus rarement à l’âge adulte, et qui exige généralement une correction chirurgicale. Les données sur les séquelles à long terme sont limitées.

**Méthodologie:**

Nous avons passé en revue les dossiers de tous les patients ayant reçu un diagnostic d’ANCG au sein de notre système hospitalier entre 1965 et 2022. Les patients qui présentaient d’autres cardiopathies structurales ont été exclus.

**Résultats:**

Trente-et-un patients ont été retenus, dont 9 (29,0 %) ont reçu leur diagnostic à l’âge adulte. La cohorte a été divisée en deux sous-groupes, soit le sous-groupe pédiatrique (< 18 ans) et celui des adultes (≥ 18 ans). L’insuffisance cardiaque a été la manifestation la plus fréquente dans le sous-groupe pédiatrique (36,4 %), alors que chez les adultes, la dyspnée et la douleur thoracique ont été plus fréquentes (44,4 % et 33,3 %, respectivement). Tous les patients ont subi une réparation chirurgicale. La survie globale (SG) à 1 an et à 5 ans, était de 93,4 % (intervalle de confiance [IC] à 95 % : 85-100) tandis que la SG à 10 ans était de 83,6 % (IC à 95 % : 69,8-100). Le taux de réintervention était de 0 % après 1 an, de 15,8 % après 5 ans (IC à 95 % : 0-31) et de 29,8 % après 10 ans (IC à 95 % : 3,4-49). La fraction d’éjection (fraction d’éjection du ventricule gauche [FEVG]) préopératoire était de 51 % dans le sous-groupe pédiatrique et de 43 % chez les adultes. Au moment du suivi, la FEVG était de 61 % dans le sous-groupe pédiatrique et de 55 % chez les adultes. L’échocardiogramme préopératoire a révélé une insuffisance mitrale marquée chez 30,8 % des patients pédiatriques et chez 42,9 % des adultes. Lors du suivi à long terme, la prévalence de l'insuffisance mitrale avait diminué pour atteindre 11,8 % dans le sous-groupe pédiatrique, toutefois, elle atteignait toujours 40 % chez les adultes.

**Conclusions:**

La présentation de l’ANCG varie chez l’enfant et l’adulte. Le taux de SG après l’intervention chirurgicale est excellent. Une amélioration de la FEVG est observée dans l’ensemble de la cohorte et on note une amélioration de l'insuffisance mitrale dans le sous-groupe pédiatrique. Il est à noter que le taux de réintervention augmente au fil du temps.

Anomalous left coronary artery (LCA) from the pulmonary artery (ALCAPA) is a rare but severe congenital cardiac anomaly that has an estimated incidence of 1 in 300,000 live births, corresponding to 0.25%-0.5% of all congenital heart disease.^1^ In this condition, the LCA originates abnormally from the pulmonary artery instead of the aorta. During pregnancy, fetuses with ALCAPA are asymptomatic due to similar diastolic pressures in the pulmonary artery and the aorta in prenatal circulation.

**Figure 1:**
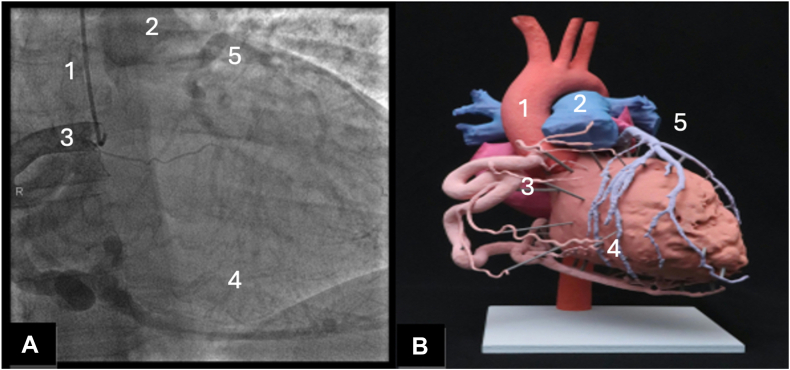
(**A**) Coronary angiography demonstrating a dilated RCA collateralizing the left coronary arterial system, a typical finding in patients with ALCAPA syndrome. (**B**) Three-dimensional printed anatomic model of the heart of a patient with ALCAPA for surgical planning purposes, demonstrating the same large collateralized RCA system. Collateralization in this case leads to left-to-right shunting to the pulmonary artery through the anomalous left coronary artery. (1) aorta, (2) main pulmonary artery, (3) dilated RCA, (4) collateralized vessels, and (5) anomalous LCA. ALCAPA, anomalous left coronary artery from the pulmonary artery; LCA, left coronary artery; RCA, right coronary artery.

After birth, however, the drop in pulmonary vascular resistance causes symptoms in most infants because of flow reversal through the LCA.^2^ In addition, collaterals begin to form between the LCA and right coronary artery, resulting in coronary artery steal secondary to left-to-right shunting.^2^ Without sufficient collateralization, these alterations lead to significant myocardial ischemia and left ventricular dysfunction^3^ (Fig. 1). Over time, mitral valve regurgitation (MR) may occur because of ventricular dilation and papillary muscle ischemia. Left untreated, infants with ALCAPA have a mortality rate of 90% during their first year.^3^ Those who do survive beyond infancy show evidence of extensive collateral arteries, although these patients may have underlying subendocardial ischemia that promotes sudden cardiac death secondary to ventricular arrhythmias.^4^

Definitive treatment for ALCAPA is surgical correction with the goal of re-establishing a dual coronary artery system, which significantly improves prognosis.^5^ Surgical techniques include direct reimplantation of the LCA into the aorta, creating a bypass graft, intrapulmonary artery baffle (Takeuchi procedure), and ligation of the LCA.^6^, ^7^, ^8^ The Takeuchi procedure is used when the length of the anomalous LCA is insufficient for a tension-free connection to the aorta.^8^ Instead of direct reimplantation, an intrapulmonary baffle is created using a pulmonary arterial flap, connecting the LCA to the aorta via the baffle. The pulmonary artery is then repaired using pericardial tissue. This technique carries a small but substantial risk of supravalvular pulmonary stenosis.^7^, ^8^ Although there is consensus regarding the superiority of anatomic repair with coronary translocation, many patients have undergone the various repair techniques used historically, and there remains uncertainty about the necessity of concomitant mitral valve repair.^5^, ^6^, ^7^ Restoring the true anatomy results in a rapid recovery of left ventricular function within the first year after surgery.^5^^,^^9^ Ventricular remodeling alone could result in significant improvement of MR secondary to ALCAPA.^9^

Although surgical repair of ALCAPA has shown improvement in early outcomes, late complications such as coronary insufficiency, MR, and heart failure have been reported. These issues may necessitate additional interventions and can impact long-term prognosis.^10^^,^^11^ There are limited data regarding long-term outcomes after ALCAPA repair.^2^^,^^7^^,^^12^, ^13^, ^14^ The objective of our study was to review our experience with ALCAPA repair, specifically evaluating the long-term outcomes of left ventricular function and mitral valve function.

## Methods

All patients with ALCAPA seen at all Mayo Clinic sites from 1965 to 2022 were retrospectively reviewed. Patients were excluded if they had other structural heart disease.

Continuous variables were summarized using median and interquartile range [IQR], and categorical variables were summarized using frequency and percentage. We retrospectively reviewed all patients with ALCAPA who were seen at our hospital system from 1965 to 2022. The study was approved by the institutional review board within our hospital system.

Patient demographics, comorbidities, operative notes and type of surgical repair, echocardiography, and angiography reports were reviewed. Patients were excluded if they had other structural heart disease. We used age at ALCAPA repair to define 2 age groups: pediatric patients (<18 years) and adults (>18 years). The left ventricular function was by transthoracic echocardiography using Simpson's biplane volumetric assessment of left ventricular ejection fraction (LVEF) in both apical 4-chamber and 2-chamber views. MR was assessed using Doppler echocardiography and graded using conventional guidelines: 0—none, 1—mild, 2—moderate, 3—moderate-severe, and 4—severe.^15^ Transthoracic echocardiography parameters were followed longitudinally. Time points included the preoperative echo, postoperative period (any images before discharge from the same admission after surgery), midterm follow-up (images obtained approximately 1 year after surgery), and long-term follow-up (most recent echocardiogram >1 year after surgery).

Continuous variables were summarized using the mean and standard deviation of normal distributions or median and IQR of skewed distributions. Frequencies were summarized as absolute numbers and percentages. A Kaplan-Meier curve was used to estimate overall survival and freedom from reintervention. Logistic regression was used to evaluate the relationship between LVEF and mortality. Patients were censored as of the date of the last follow-up. A *P* value of <0.05 was considered to be significant. Statistical software R 4.1.2 was used for analysis.

## Results

A total of 31 patients were identified, and baseline demographics are shown in Table 1. The pediatric subgroup consisted of 22 patients (31.8% male) compared with 9 patients in the adult subgroup (33.3% male). The median age at diagnosis was 0.0 (IQR: 0.0-5.5) years in pediatric patients and 47 (IQR: 41.5-49) years in adults, with similar median ages at the time of intervention (pediatric patients 0.5 years, adults 48 years). Heart failure was the most common presentation in pediatric patients (36.4%), followed by dyspnea (27.3%). Presenting symptoms in adults included dyspnea (44.4%), chest pain (33.3%), heart failure (11.1%), arrhythmia (11.1%), dizziness (11.1%), and palpitations (11.1%), with 5 of the patients reporting more than 1 symptom at presentation.

**Table 1:** Baseline demographics and clinical characteristics of the study cohort—patients treated for isolated ALCAPA at Mayo Clinic between 1965 and 2022

	Pediatric (n = 22)	Adult (n = 9)	Total (N = 31)
Sex, n (%)			
Male	7 (31.8)	3 (33.3)	10 (32.3)
Female	15 (68.2)	6 (66.7)	21 (67.7)
Age at diagnosis			
Median (IQR)	0.0 (0.0, 5.5)	47.0 (41.5, 49.0)	5.5 (0.0, 35.8)
Missing	7	2	9
Age at intervention			
Median (IQR)	0.5 (0.0, 7.8)	48.0 (41.0, 50.0)	7.0 (0.0, 27.0)
Length of follow-up (mo)			
Median (IQR)	105.6 (35.9, 250.4)	22.1 (10.5, 70.3)	79.5 (10.5, 198.6)
Missing	1	0	1
Presenting symptoms, n (%)			
Chest pain	1 (4.5)	3 (33.3)	4 (12.9)
Dyspnea	6 (27.3)	4 (44.4)	10 (32.3)
Heart failure	8 (36.4)	1 (11.1)	9 (29.0)
Myocardial infarction	0 (0.0)	0 (0.0)	0 (0.0)
Arrhythmia	0 (0.0)	1 (11.1)	1 (3.2)
Dizziness	2 (9.1)	1 (11.1)	3 (9.7)
Palpitations	2 (9.1)	1 (11.1)	3 (9.7)
Loss of consciousness	0 (0.0)	0 (0.0)	0 (0.0)
Mode of diagnosis			
Imaging, n (%)	14 (100.0)	7 (100.0)	21 (100.0)
Missing	8	2	10
Surgical intervention, n (%)			
Ligation and CABG	3 (13.6)	2 (22.2)	5 (16.1)
Direct reimplantation	9 (30.9)	7 (77.8)	16 (51.6)
Takeuchi procedure	6 (27.3)	0 (0.0)	6 (19.4)
Other	4 (18.2)	0 (9.1)	4 (12.9)
Comorbidities, n (%)			
Hypertension	0 (0.0)	2 (22.2)	2 (6.5)
Diabetes	0 (0.0)	0 (0.0)	0 (0.0)
Hyperlipidemia	0 (0.0)	3 (333)	3 (9.7)
Sleep apnea	0 (0.0)	2 (22.2)	2 (6.5)
Tobacco use	0 (0.0)	2 (22.2)	2 (652)
Arrhythmia	2 (9.1)	4 (44.4)	6 (19.4)
CVA/TIA	0 (0.0)	1 (11.1)	1 (3.2)
PE/DVT	1 (4.5)	0 (0.0)	1 (3.2)

ALCAPA, anomalous left coronary artery from the pulmonary artery; CABG, coronary artery bypass grafting; CVA, cerebrovascular accident; DVT, deep vein thrombosis; IQR, interquartile range; PE, pulmonary embolism; TIA, transient ischemic attack.

None of the patients in our study underwent simple ligation. The most common method of surgical correction was the direct reimplantation technique (pediatric patients 40.9% and adults 77.8%). Among pediatric patients, 6 (27.3%) underwent the Takeuchi procedure, which was not used in any adult. The median intensive care unit length of stay was 2.5 days, and the mean hospital stay was 5.5 days.

Overall survival was 93.4% at 1 year (95% confidence interval [CI]: 85-100), 93.4% at 5 years (95% CI: 85-100), and 83.6% at 10 years (95% CI: 69.8-100), as shown in Table 2. Stratified by subgroup, overall survival was 90.7% (95% CI: 79.2-100) at years 1, 5, and 10 in pediatric patients compared with 100% at years 1 and 5, and 60% (95% CI: 29.3-100) at year 10 in adults respectively (*P* = 0.116). All of the patients in the study underwent some form of surgical corrective procedure, and none experienced sudden cardiac death before intervention. Of the 31 patients in the study, 8 were deceased at the time the study was conducted. Two of these patients died during or shortly after surgery (within 1 day) and were both in the pediatric group. The other 6 were in the adult subgroup and died 5-40 years after their initial surgery; details on the cause of death for these patients were not available.

**Table 2:** Ninety-five percent confidence intervals of ALCAPA patients’ overall survival rates and freedom from reintervention, stratified by age cohort

	1 year	5 years	10 years
Overall survival (%)			
Overall	93.4 (85-100)	93.4 (85-100)	83.6 (69.8-100)
Pediatric patients	90.7 (79.2-100)	90.7 (79.2-100)	90.7 (79.2-100)
Adults	100 (100-100)	100 (100-100)	60 (29.3-100)
Need for reintervention (%)			
Overall	0 (0-0)	15.8 (0-31)	29.8 (3.4-49)
Pediatric patients	0 (0-0)	18.8 (0-35.8)	33.5 (4.3-53.8)
Adults	0 (0-0)	0 (0-0)	0 (0-0)

**Figure 2:**
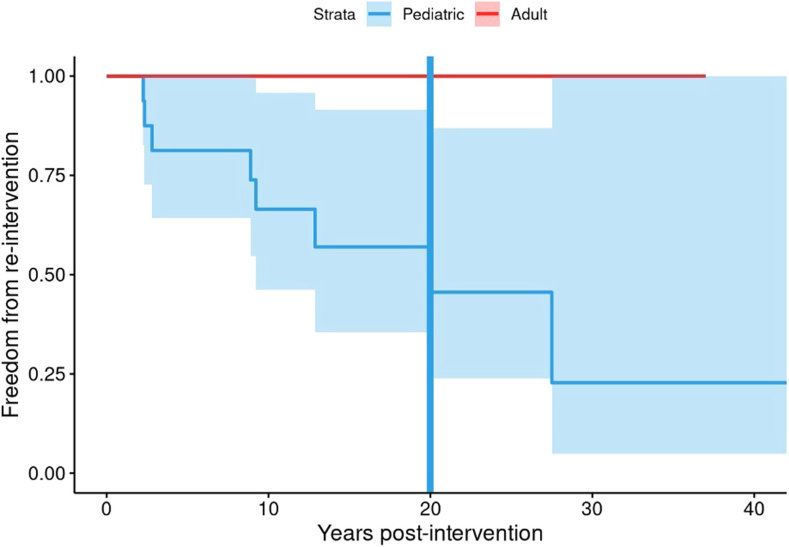
Kaplan-Meier curve showing estimate of freedom from reintervention after the initial anomalous left coronary artery from the pulmonary artery corrective procedure. Fifty percent of patients required reintervention at 20 years after the initial procedure; this was driven by reintervention in the pediatric cohort entirely (**blue**). The adult cohort (**red**) did not have any reintervention occurrences.

The need for reintervention for the whole sample was 0% at 1 year, 15.8% at 5 years (95% CI: 0-30.7), and 29.8% at 10 years (95% CI: 3.4-49), driven entirely by pediatric patients. When considering only pediatric patients, the need for reintervention for 1 year was 0%, 18.8% (95% CI: 0-35.8) at 5 years, and 33.5% (95% CI: 4.3-53.8) at 10 years. No reintervention was performed in adults (Table 2). Figure 2 demonstrates a Kaplan-Meier curve of estimated freedom from reintervention over time, with 50% for the whole sample estimated needing reintervention at year 20 (Fig. 2). Reasons for reintervention often included occlusion or stenosis of the reimplanted left main coronary artery or Takeuchi baffle. The types and nature of reintervention performed are shown in Table 3.

**Table 3:** Timing and nature of reintervention

Age at intervention	Initial surgical intervention	Age at reintervention	Nature of reintervention
0	Direct reimplantation with Gore-Tex graft	9	Occlusion of Gore-Tex graft reimplanted LM requiring redo sternotomy with CABG LIMA to LAD
0	Reimplantation via 4-mm interposition Gore-Tex graft	9	Graft occlusion requiring redo sternotomy for CABG
0	Ligation and CABG	20	Right subclavian to LAD CABG transected during the mitral commissurotomy procedure, need for redo CABG with placement of SVG to LAD
0	Takeuchi procedure	2	Takeuchi repair LM stenosis, main pulmonary artery stenosis, and right pulmonary artery stenosis. Redo sternotomy for Takeuchi revision, main PA patch, and RPA stenting
1	Takeuchi procedure	14	Takeuchi baffle leak and stenosis. Redo sternotomy with ligation and CABG (LIMA to LAD)
2	Takeuchi procedure	4	Takeuchi repair stenosis and mitral regurgitation. Redo sternotomy with Gore-Tex patch repair of baffle stenosis and mitral valve annuloplasty
7	Takeuchi procedure	35	Baffle leak and large pseudoaneurysm and pulmonary artery and vein compression. Redo sternotomy with CABG (LIMA to LAD) with ligation of Takeuchi repair
17	Direct reimplantation	19	LM stenosis. Redo sternotomy with CABG (LIMA to LAD)

CABG, coronary artery bypass grafting; LAD, left anterior descending artery; LIMA, left internal mammary artery; LM, left main; PA, pulmonary artery; RPA, right pulmonary artery; SVG, saphenous vein graft.

**Figure 3:**
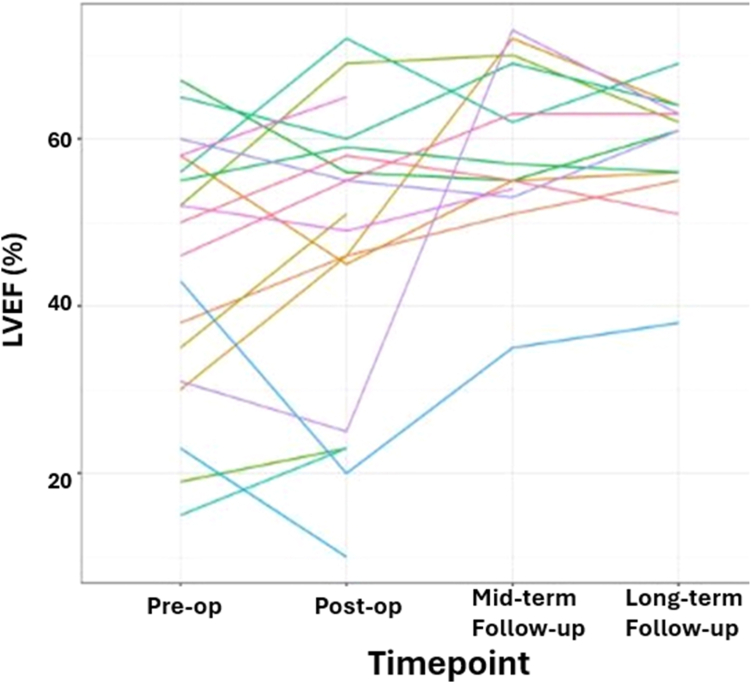
Scatter plot demonstrating left ventricular ejection fraction (LVEF) in all study participants stratified over time. There is a trend of improvement in LVEF over time after corrective surgery for anomalous left coronary artery from the pulmonary artery syndrome. There was no statistically significant relationship between LVEF and mortality in the entire study at each time point (Table 5).

The median preoperative LVEF for pediatric patients was 51% (IQR: 30.8-58) compared with 55% (IQR: 45-65) early postoperatively, 62% (IQR: 55-70) at midterm follow-up, and 61% (IQR: 57.5-62.2) at long-term follow-up. LVEF for adults was 43% (IQR: 36.5-53.5) preoperatively compared with 49% (IQR: 34.5-55) early postoperatively, 54% (IQR: 51-57) at midterm follow-up, and 55% (IQR: 48-56) for adults at long-term follow-up, as seen in Table 4. For the entire cohort, the median LVEF was 50% (IQR: 33-57) preoperatively, which improved to 56% (IQR: 54.2-67.5) at midterm follow-up. Recovery was sustained at long-term follow-up, with a median ejection fraction of 60% (IQR: 56-62). Logistic regression showed no relationship of mortality to preoperative LVEF (*P* = 0.205), or to postoperative (*P* = 0.151), midterm follow-up (*P* = 0.429), or long-term follow-up LVEF (*P* = 0.079) (Table 5). The scatter plot of all patients' LVEF over time is shown in Figure 3.

**Table 4:** Left ventricular ejection fraction and rates of mitral regurgitation over time

Age at intervention	Preoperatively (n = 31)	Postoperatively (n = 31)	Midterm follow-up (n = 31)	Long-term follow-up (n = 31)
LVEF, median (IQR)				
Overall (N = 31)	50 (33, 57)	53 (40, 59.2)	56 (54.2, 67.5)	60 (56, 62)
Missing	12	11	17	10
Pediatric patients	51 (30.8, 58)	55 (45, 65)	62 (55, 70)	61 (57.5, 62.2)
Missing	10	9	13	6
Adults	43 (36.5, 53.5)	49 (34.5, 55)	54 (51, 57)	55 (48, 56)
Missing	2	2	4	4
MR, frequency (%)				
Overall (N = 31)	7 (35)	4 (21.1)	2 (15.4)	4 (18.2)
Missing	11	12	18	9
Pediatric patients	4 (30.8)	3 (25)	2 (25)	2 (11.8)
Missing	9	10	14	5
Adults	3 (42.9)	1 (14.3)	0 (0)	2 (40)
Missing	2	2	4	4

IQR, interquartile range; LVEF, left ventricular ejection fraction; MR, mitral regurgitation.

**Table 5:** Logistic regression results for left ventricular ejection fraction at each time period related to mortality

Time point	Odds ratio	(95% CI)	*P* value
Preoperative	0.93	(0.81-1.03)	0.205
Postoperative	0.93	(0.82-1.01)	0.151
Mid-term follow-up	0.92	(0.74-1.14)	0.429
Long-term follow-up	0.84	(0.66-1)	0.079

CI, confidence interval.

**Figure 4:**
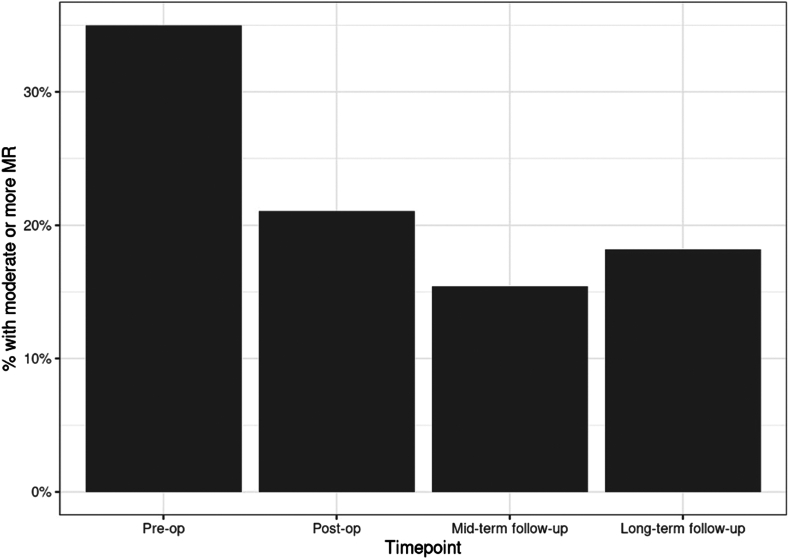
Percentage of patients noted to have moderate or more mitral regurgitation (MR) on an echocardiogram over time. Rates of moderate or more MR consistently dropped at each time point in pediatric patients. Overall rates of moderate or more MR in the entire study were 35% preoperatively, 21.1% postoperatively, 15.4% at midterm follow-up, and 18.2% at long-term follow-up.

Moderate or more MR was noted in preoperative echocardiograms in 30.8% of pediatric patients and 43% of adults. This improved to 25% at postoperative and midterm follow-up time points and 11.8% at long-term follow-up in pediatric patients. Moderate or more MR was present in 42.9% preoperatively in adults and improved to 14.3% postoperatively and 0% at midterm follow-up but was 40% in adults at long-term follow-up (see Table 4 and Fig. 4). Overall rates of moderate or more MR in the entire study were 35% preoperatively, which improved to 21.1% postoperatively, 15.4% at midterm follow-up, and 18.2% at long-term follow-up.

## Discussion

ALCAPA is a rare but life-threatening congenital coronary artery anomaly. Diagnosis is typically made through imaging modalities such as echocardiography and computed tomography or, less commonly, by invasive angiography.

Young patients require prompt surgical correction irrespective of symptom burden or extent of intercoronary collateralization.^16^ Over time, the surgical approach to ALCAPA repair has evolved, with the reimplantation technique now widely adopted as a standard procedure for the majority of patients. Not surprisingly, most of our patients had direct reimplantation as their surgical procedure, while some patients had undergone the Takeuchi procedure or ligation and coronary artery bypass grafting. The latter likely had their surgical corrections performed in a period previous to the current standard, as this study population spanned several decades. By 10 years postoperatively, 8 patients (38%) required reintervention, all of whom had their initial intervention in the pediatric age range (pediatric patients).

The Takeuchi procedure has known complications that include baffle leaks, baffle obstruction, and supravalvular pulmonary artery stenosis.^17^ Four of the 6 patients who underwent initial repair with the Takeuchi procedure underwent reintervention due to the development of 1 or more of these complications. Three patients had complications related to an initial direct reimplantation technique, with or without use of grafting material, all of which had developed stenosis and required redo sternotomy with coronary artery bypass grafting.

Early postoperative recovery was excellent, with a mean length of stay in the intensive care unit of 2.5 days and a mean hospital stay of 5.5 days. These durations are shorter than those reported in other studies.^1^^,^^2^ Only one of our patients (3%) required preoperative mechanical ventilation; however, other studies noted that up to one-third of their patients required preoperative mechanical ventilation.^11^ Three patients (9.6%) required extracorporeal membrane oxygenation, which is similar to what is reported by other groups.^18^^,^^19^

Our study showed favorable overall outcomes, with excellent short- and long-term survival after initial ALCAPA repair. Overall survival rates were 93.4% at 1 and 5 years and 83.6% at 10 years. This is in keeping with the published data regarding this condition and its repair procedures.^2^^,^^5^, ^6^, ^7^ Among our entire cohort, there was an improvement in the LVEF after surgical intervention. Recovery was sustained at long-term follow-up, with a mean LVEF of 60%. LVEF was not associated with late patient outcomes in this study, likely due to the small number of patients. However, LVEF is often followed longitudinally due to its prognostic value in predicting mortality in patients with heart failure, and a positive trend suggests some improvement in cardiac function over time after corrective surgery.

Seven patients (35%) initially presented with moderate or more MR, which improved postoperatively in most patients. There is an ongoing debate regarding the need for repair of severe MR during the initial repair of ALCAPA. Our study and others^5^ have shown significant improvement in mitral valve function after repair. Isomatsu et al.^11^ compared 2 groups of 29 patients with ALCAPA and noted that additional mitral valve repair did not impact postoperative outcomes or influence ventricular systolic function after repair. This is consistent with the concept that the primary cause of MR in patients with ALCAPA is predominantly functional, stemming from left ventricular ischemia-induced annular dilation,^2^^,^^5^ and with revascularization and ventricular remodeling, both left ventricle and mitral valve function generally improve.^5^^,^^9^^,^^20^ Although some experts recommend performing mitral valve annuloplasty for all patients with severe MR during ALCAPA repair to mitigate the risk of postoperative hemodynamic instability and avoid the need for future mitral valve repair,^11^^,^^21^ mitral valve repair may not be straightforward in young patients, and adding the procedure should be balanced against a possible increase in early surgical risk.^9^^,^^20^ Based on these data, our institution does not typically intervene on the mitral valve during the initial ALCAPA repair unless there is a structural valve defect as was seen in one patient in our study. Only 2 patients (6.4%) required reoperation on the mitral valve after ALCAPA repair.

### Limitations

This study shares the typical limitations of a retrospective study, and it included only patients seen at Mayo Clinic sites; the results may not be generalizable. Although, to our knowledge, we present one of the largest cohorts of patients with ALCAPA, the study's sample size remains very small, limiting data analysis and interpretation. Further, late echocardiographic data are not complete for all patients.

## Conclusions

ALCAPA, a rare, life-threatening congenital coronary anomaly requiring surgical correction, has variable presentations in childhood and adulthood. The current study demonstrates long-term outcomes after surgery at our center between 1965 and 2022. Results show excellent overall survival, an improvement in LVEF over time throughout the cohort and MR improved in pediatric patients. The need for reintervention increased over time driven by complications in pediatric patients. There is a need for further study in this small population of patients.
